# Comparative study of layer-by-layer deposition techniques for poly(sodium phosphate) and poly(allylamine hydrochloride)

**DOI:** 10.1186/1556-276X-8-539

**Published:** 2013-12-20

**Authors:** Cesar Elosua, Diego Lopez-Torres, Miguel Hernaez, Ignacio R Matias, Francisco J Arregui

**Affiliations:** 1 Nanostructured Optical Devices Laboratory, Electric and Electronic Engineering Department, Public University of Navarra, Edif. Los Tejos, Campus Arrosadía, Pamplona 31006, Spain

**Keywords:** Layer-by-layer, Dipping and spray deposition, Inorganic polymer, Hydrophilic film, Functionalized surfaces

## Abstract

An inorganic short chain polymer, poly(sodium phosphate), PSP, together with poly(allylamine hydrochloride), PAH, is used to fabricate layer-by-layer (LbL) films. The thickness, roughness, contact angle, and optical transmittance of these films are studied depending on three parameters: the precursor solution concentrations (10^-3^ and 10^-4^ M), the number of bilayers deposited (20, 40, 60, 80, and 100 bilayers), and the specific technique used for the LbL fabrication (dipping or spraying). In most cases of this experimental study, the roughness of the nanofilms increases with the number of bilayers. This contradicts the basic observations made in standard LbL assemblies where the roughness decreases for thicker coatings. In fact, a wide range of thickness and roughness was achieved by means of adjusting the three parameters mentioned above. For instance, a roughness of 1.23 or 205 nm root mean square was measured for 100 bilayer coatings. Contact angles close to 0 were observed. Moreover, high optical transmittance is also reported, above 90%, for 80 bilayer films fabricated with the 10^-4^ M solutions. Therefore, these multilayer structures can be used to obtain transparent superhydrophilic surfaces.

## Background

Among different deposition techniques, the layer-by-layer (LbL) method has focused the attention of a large number of research groups. The pioneering work of Iler in 1966 [1] did not become public until it was rediscovered by Decher in the beginning of 1990s as a simple and automatable method to fabricate films at the nanometer scale [1,2]. Compared to LbL, other deposition techniques are limited to flat substrates and require expensive and delicate instrumentation [3]. On the contrary, LbL does not depend neither on the substrate shape or size and a wide range of different materials can be deposited on different substrates such as windows [4] or small optical fibers [5-7]. Additionally, this method can be also used to attach analytes of different chemical nature [8,9]. As a consequence of these features, LbL has been used to functionalize surfaces with different goals such as antibacterial applications [10], the fabrication of hydrophobic or hydrophilic films [11,12], or to develop sensors [13,14]. The main idea of LbL method consists of the assembly of oppositely electrically charged polyelectrolytes (polycation and polyanion respectively) which form a bilayer [15]; the process can be repeated as many times as the design requires. The chemical properties of the polyelectrolytes, such as the average molecular weight, the ionization degree, the concentration or the ionic strength [16,17], just to mention some of the most important ones, define the morphology of the final film and, hence, its features.

The polyelectrolytes that can be used are divided in two categories, the strong and weak ones: in the first group, the ionization degree is not adjustable, whereas in the second one, it is adjustable by the pH of the solution [18]. Depending on the ionization degree, the polymers get adsorbed on the substrate in a different manner: highly ionized solutions would yield to flat polyelectrolytes and very thin films; meanwhile, low ionization levels produce curled chains and rough layers [19]. As the pH can be used to set the ionization degree, typically at least one of the polymers is weak, although in most times both of them belong to this category. In the case of polyelectrolytes whose ionization degree is not adjustable, the ionic strength of the solution can be varied by adding salts, and in this manner, altering the morphology of the polymer chains by electrostatical interactions [20]. Another important factors are temperature, which defines the kinetics of the process [21], as well as the way the substrates is exposed to the polyelectrolytes solutions, for example, by dipping or spray [22].

Some of the ideas that were established about LbL, as the ones mentioned above, have been set under consideration. It was supposed the *Z* potential of the last deposited layer should always show the opposite sign of the following one; on the other hand, the roughness of the film was accepted to be reduced as the films grows. A recent work has revealed that when using certain polymers, these rules are not satisfied [23]: With a 10^-4^ M concentration of poly(sodium phosphate) (PSP) and poly(allylamine hydrochloride) (PAH), the *Z* potential is not alternated between one layer and the next one; moreover, the roughness of the film increases with the number of bilayers when the substrate is sprayed with the polymeric solutions [23]. This behavior seems to be a consequence of using PSP, an inorganic short chain polymer with interesting properties; the use of this kind of polymers establishes a new researching line and raises again some questions about the fundamentals of LbL, taking into account other non-electrostatic interactions such as hydrogen bonds during the growing process of the film [24]. In the light of these results, some works have focused in the study of the key parameters of LbL in order to revise the effect of polymers as PSP in detail and redefine the rules of this technique [24].

In this work, nanofilms were prepared onto glass slides using PSP and PAH. Two different concentrations were used for the experiments, 10^-3^ and 10^-4^ M, because these are the same concentration values reported in the sprayed films studied by Decher et al. [23]. Moreover, the substrates were dipped or sprayed with the solutions to check also how these alternatives affect the features of the film. The growing process was evaluated by preparing substrates with different number of bilayers so that their thickness, roughness, contact angle, and optical transmittance spectra were measured. To our knowledge, this is the first time that a comparative study of the properties of PSP/PAH films fabricated by dip-coating LbL and spray-assisted LbL is presented in the literature.

## Methods

### Materials

The polymers used were PAH (*M*_w_ ~ 58,000), PSP, P_2_O_5_ basis, and poly(ethylenimine) (PEI) (*M*_w_ ~ 25,000). All chemicals were purchased from Sigma-Aldrich (St. Louis, MO, USA) and used without further purification. All aqueous solutions were prepared using ultrapure water with a resistivity of 18.2 MΩ cm.

### Construction of the nanofilms

The glass slides were treated in order to eliminate any organic remains and also to enhance the hydroxyl density onto their surface. To achieve it, the slide was immersed in a solution of water and detergent, sonicating it for 10 min; thereafter, the substrate was sonicated again for the same time in ultrapure water. Finally, it was dipped into a 1 M KOH aqueous solution for 10 min and sonicated once more in ultrapure water for the same time. Between each step, the glass slide was dried with nitrogen. In order to promote the initial growing of the nanofilms, an anchoring layer was deposited onto the slides by dipping them into a 2.5 mg/1 mL of PEI aqueous solution for 10 min; thereafter, the slide was rinsed with ultrapure water for 10 min and dried with nitrogen.

Solutions with concentrations of 10^-3^ and 10^-4^ M for PAH and PSP were prepared; in all cases, the mixtures had a 0.15 M NaCl to set the ionic strength. The pH of both solutions was adjusted to 6.37 with NaOH or HCl [23].

The nanofilms were developed by either dipping the substrate into the 10^-3^/10^-4^ M solutions or by spraying the different solutions on the substrate. Therefore, up to four different growing conditions were studied (10^-3^ and 10^-4^ M of LbL dipping and 10^-3^ and 10^-4^ M of spray-assisted LbL). The anchoring layer of PEI led a positive superficial density charge onto the fiber so that each bilayer shows the structure PSP/PAH. Films with 20, 40, 60, 80, and 100 bilayers were prepared in each growing configuration in order to study the effects of the construction parameters.

In the case of the dipping process, each construction cycle was performed by immersing the slide into the PSP solution for 2 min and then rising it in ultrapure water for 1 min; thereafter, it was dipped into the PAH mixture for 2 min and rinsed again for 1 min in ultrapure water. This process was repeated as many times as required for the film. The steps were similar for the spray technique: the polymeric solutions and ultrapure water were sprayed for 10 s onto the slides. Both methods were automated by using a robotic arm (in the case of the dipping construction) and a spraying robot (both of them acquired from Nadetech Innovations S.L., Sarriguren, Spain).

### Characterization

The films prepared were characterized in order to study the growing process depending on the construction conditions. One of the key parameters, roughness, was measured by an atomic force microscope (AFM) (Veeco Innova, model 840-012-711; Veeco Instruments, Inc., Plainview, NJ, USA) in tapping mode; it was also used to register the thickness of the films by scratching the surface with a needle and scanning the cantilever perpendicularly to the scratch. For each sample, the AFM measurements were performed seven times in different zones to get the mean value and the standard deviation. AFM images were obtained by scanning 5 μm × 5 μm areas with 512 lines at a 0.1-Hz frequency. UV/Visible transmission spectra were recorded by a spectrometry transmission configuration, placing the glass slide under study in a holder between a white light source (HL2000; OceanOptics, Dunedin, FL, USA) and a spectrometer (USB2000XR1, OceanOptics). Finally, the contact angle was registered using a contact angle meter (KSV Instruments goniometer; Espoo, Finland) for each sample.

## Results and discussion

As it was cited before, four sets of samples were prepared: 10^-3^ and 10^-4^ M of LbL dipping as well as 10^-3^ and 10^-4^ M of spray-assisted LbL. In each set, five slides were coated with different number of bilayers (20, 40, 60, 80, and 100). The information will be presented in the next two sections depending on the way the glass slides are exposed to the polyelectrolyte solutions; in each section, results with different polymer concentrations are also commented.

### LbL dipping approach

**Figure 1:**
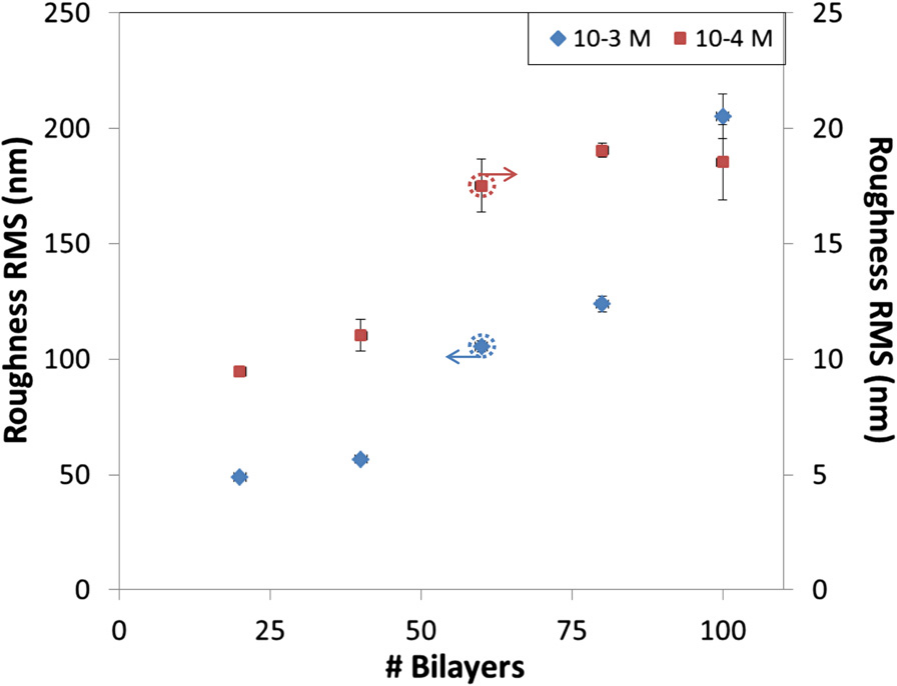
**AFM images for the films obtained when the glass slides are dipped into the 10**^**-4**^ **M solutions.** 20 bilayers **(a)**, 40 bilayers **(b)**, 60 bilayers **(c)**, 80 bilayers **(d)**, and 100 bilayers **(e)**.

**Figure 2:**
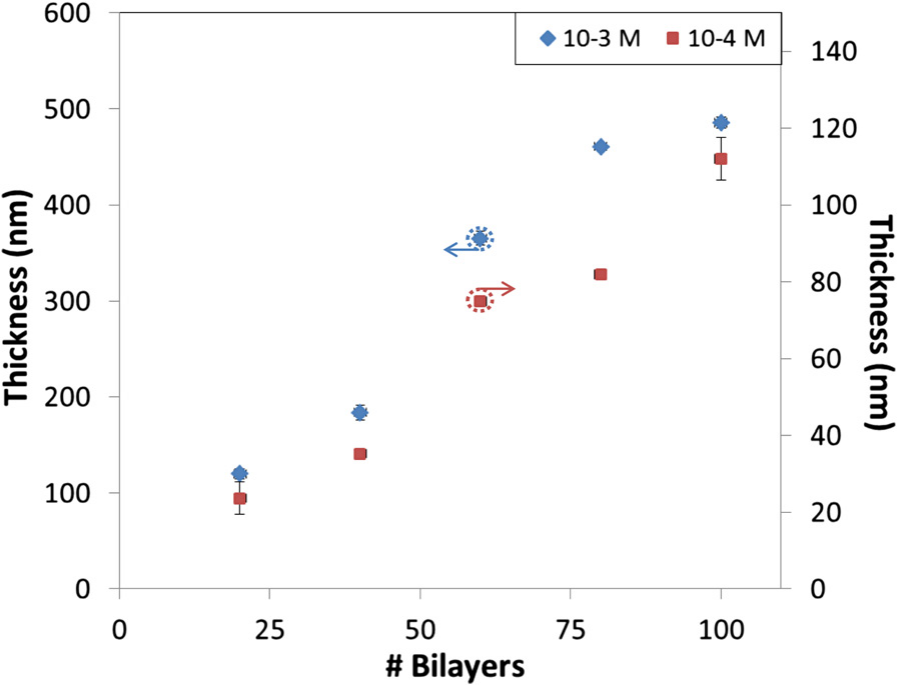
**AFM images for the films obtained when the glass slides are dipped into the 10**^**-3**^ **M solutions.** 20 bilayers **(a)**, 40 bilayers **(b)**, 60 bilayers **(c)**, 80 bilayers **(d)**, and 100 bilayers **(e)**.

**Figure 3:**
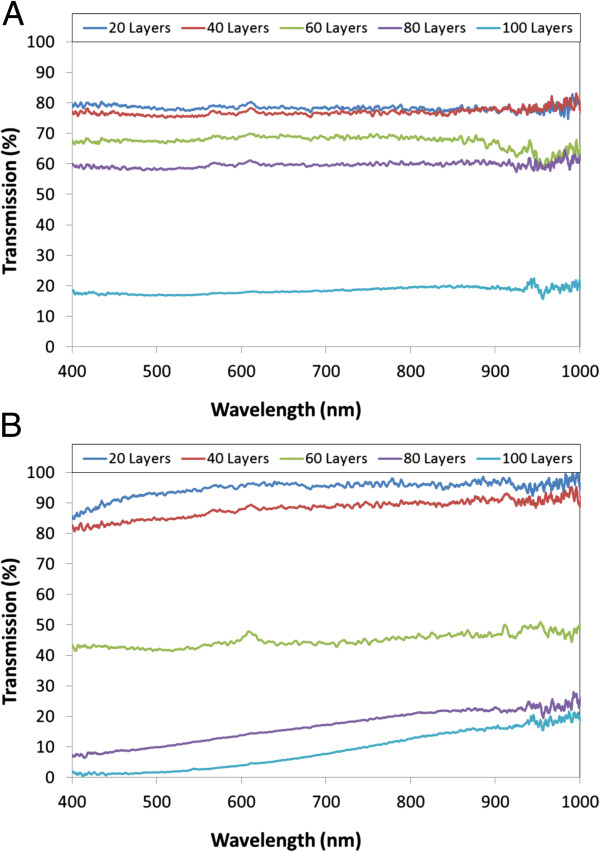
**Roughness RMS registered for the dipped glass slides.** The left vertical axe is applied for the 10^-3^ M solutions and the right vertical axe for the 10^-4^ M ones.

A traditional assumption in LbL films is that the thickness of the film increases as the number of bilayers does, whereas the root mean square (RMS) roughness decreases [25]. In order to study this statement, the first set of slides was prepared with 10^-4^ M polymer solutions (0.15 M NaCl): the AFM images obtained for 20, 40, 60, 80, and 100 bilayer films are shown in Figure 1. It can be observed that the RMS roughness increases with the number of bilayers, from 9.47 up to 18.53 nm RMS for 20 and 100 bilayers, respectively. Although this surprising behavior was reported recently for sprayed-assisted LbL coatings [23], this is the first time that it is reported for PSP/PAH films fabricated by LbL dip coating. The morphology of the films looks islandlike for the 20 bilayer films: as the number of construction cycles grows, so does the size of the island, as well as the RMS roughness. This behavior was observed in other work focused on nanostructures based on PSP [23]. The use of a short-chain inorganic polymer as PSP seems to alter the growth of the nanofilms, keeping the roughness increasing with the number of bilayers. In the case of the films prepared with 10^-3^ M solutions (Figure 2), the behavior is similar: the roughness goes from 48.98 up to 205.53 nm RMS for 20 and 100 bilayers, respectively. The morphology looks granulated in all cases, with a bigger granulate size as the number of bilayers increases. The values registered for the RMS roughness are much higher than the ones observed with 10^-4^ M solutions and also contradict what is expected from LbL films. Figure 3 shows a graph with the registered RMS roughness as a function of the number of bilayers for the slides prepared for the two concentrations; although the scale is not the same, the increasing trend is similar in both cases, which highlights the fact that PSP alters the growing of LbL films.

**Figure 4:**
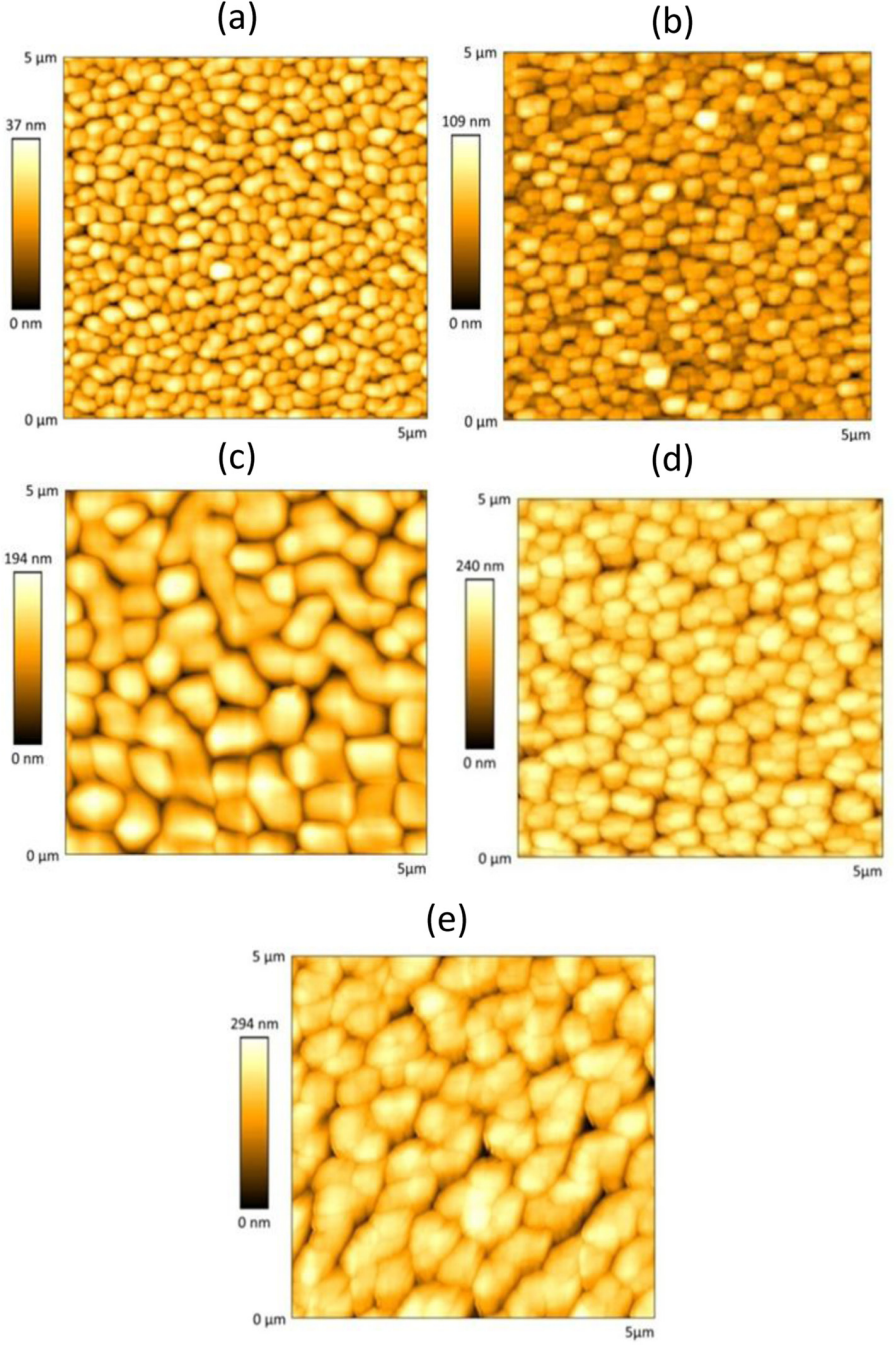
**Thickness recorded for the dipped glass slides.** The left vertical axe is applied for the 10^-3^ M solutions and the right vertical axe for the 10^-4^ M ones.

On the other hand, the thickness of the fabricated films points that the growth increases with the number of bilayers, as it can be checked in Figure 4. The thickness values obtained for the more concentrated solution are around six times higher than for the nanoconstructions prepared with the 10^-4^ M mixtures; in both cases, the thickness grows monotonically [21].

Additionally, the contact angle of the samples was also measured to study the hydrophilicity of the films [26]. In the case of the films prepared with the 10^-4^ M solutions, as a consequence of the increasing roughness with the number of bilayers, the contact angle lowers from 60° down to 28°; despite of this decrease, the films are far from being superhydrophilic. On the contrary, contact angles registered for the films prepared with the 10^-3^ M solutions are close to 0 even for 20 bilayers, which enables the utilization of these films in superhydrophilic applications [26]. Registered images of the contact angle are available in the Additional file 1.

**Figure 5:**
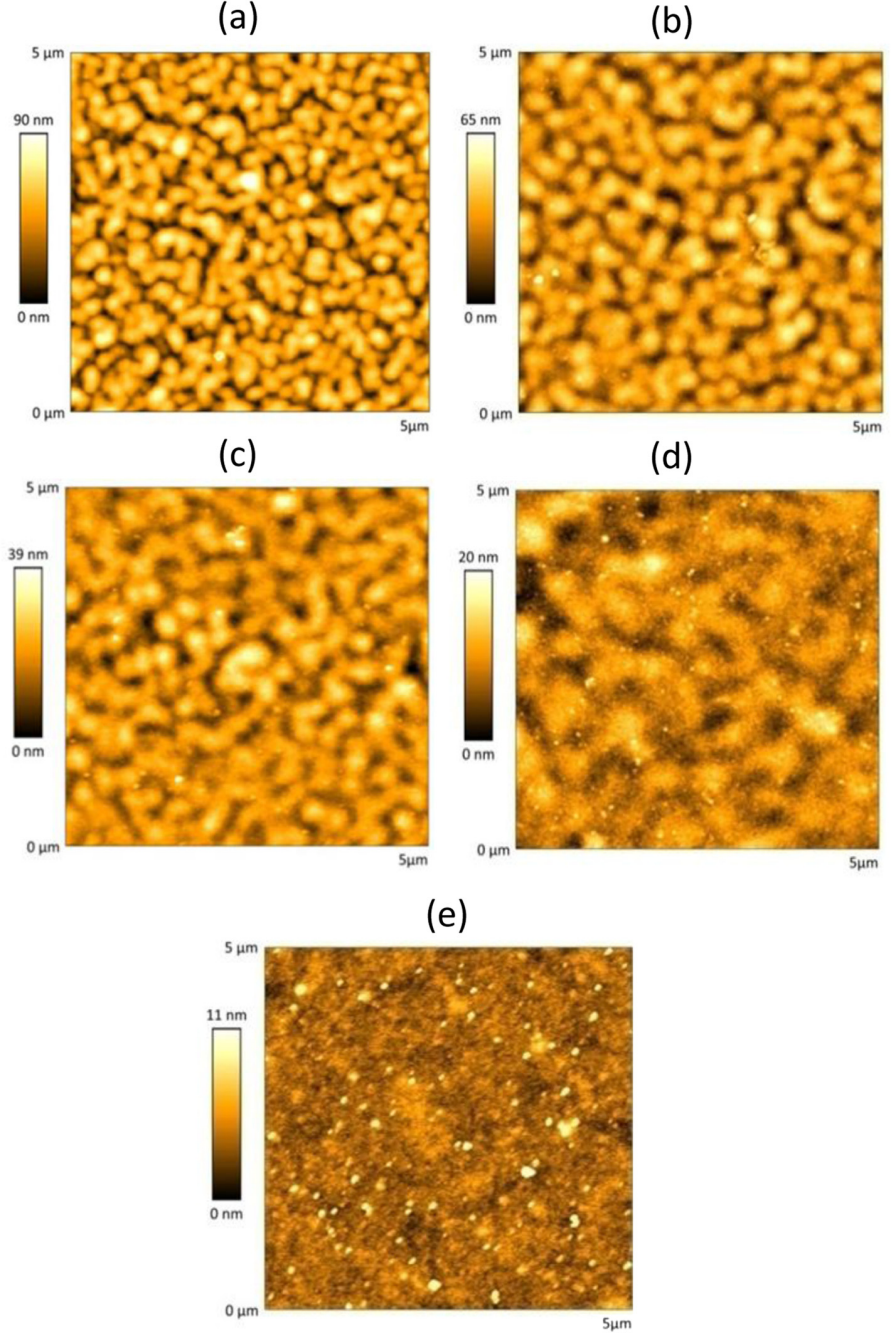
**Transmission spectra of the films developed using dipping approach.** Transmission spectra measured for the films developed using the dipping approach with the 10^-4^ M solutions **(a)** and the 10^-3^ M mixtures **(b)**.

Regarding to the transmittance spectra, the optical losses increased with the number of bilayers: in the case of 10^-4^ M prepared films, transmittance is about 80% for 20 and 40 bilayers, decreasing around 65% for 60 and 80 bilayers, and falling down to 20% in the case of the 100 bilayer films. For the other set of slides, the 10^-3^ M prepared films, the optical transmittance falls in the case of 60 bilayers and down to 15% when 100 bilayers are deposited. These results are a consequence of the increasing thickness, which is around 600 μm in the case of the film formed by 100 bilayers of the second set; the roughness could also contribute to the scattering of light, increasing the optical transmission losses. The spectra recorded are plotted in Figure 5. All the data registered are summarized in Table 1.

**Table 1:** Characterization of the films prepared using dipping approach

**Number of bilayers**	**Roughness**				**Thickness**				**Contact angle**			
	**10**^**-4**^ **M**		**10**^**-3**^ **M**		**10**^**-4**^ **M**		**10**^**-3**^ **M**		**10**^**-4**^ **M**		**10**^**-3**^ **M**	
	**μ**	**σ**	**μ**	**σ**	**μ**	**σ**	**μ**	**σ**	**μ**	**σ**	**μ**	**σ**
20	9.47	0.15	48.98	1.33	23.67	4.24	120.33	5.34	48.75	1.49	0.36	0.21
40	11.03	0.695	56.78	1.45	35.33	0.71	184.12	7.78	65.50	1.55	3.31	0.81
60	17.51	1.16	105.5	2.34	75.11	1.41	365.03	7.07	30.12	0.91	0	0
80	19.05	0.29	123.93	3.51	82.07	0.70	461.06	0.35	28.51	1.66	0	0
100	18.53	1.62	205.23	9.79	112.02	5.65	486.07	5.65	28.02	1.41	0	0

### Spray-assisted LbL approach

**Figure 6:**
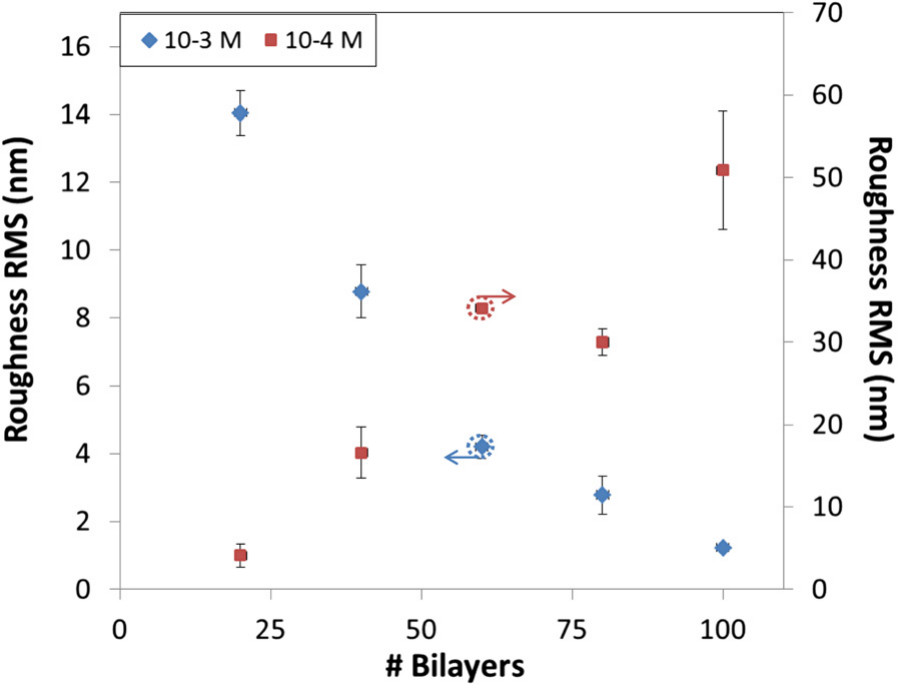
**AFM images for the films obtained when the glass slides are sprayed into the 10**^**-4**^ **M solutions.** 20 bilayers **(a)**, 40 bilayers **(b)**, 60 bilayers **(c)**, 80 bilayers **(d)**, and 100 bilayers **(e)**.

**Figure 7:**
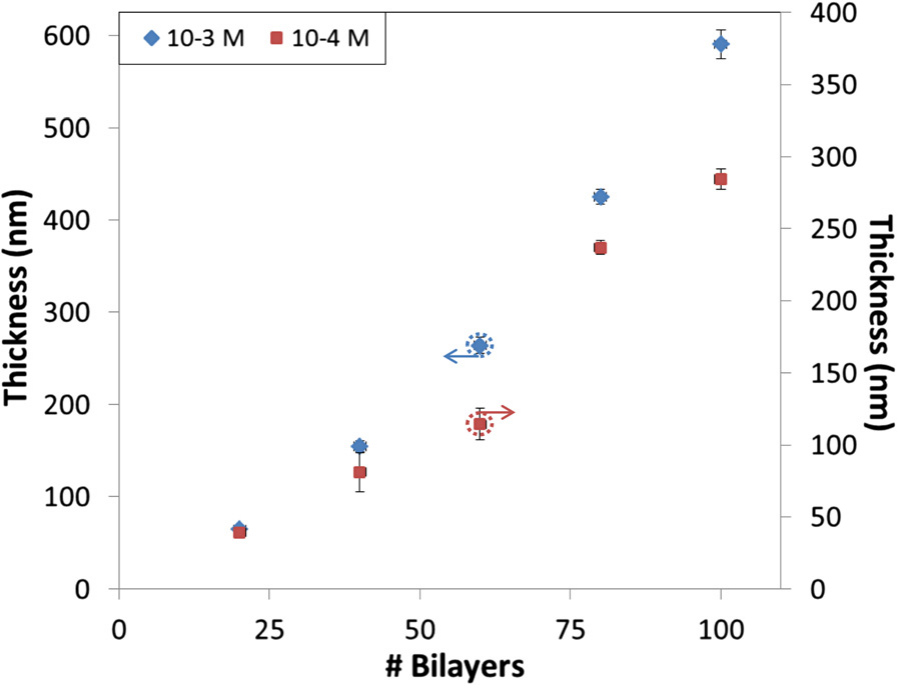
**AFM images for the films obtained when the glass slides are sprayed into the 10**^**-3**^ **M solutions.** 20 bilayers **(a)**, 40 bilayers **(b)**, 60 bilayers **(c)**, 80 bilayers **(d)**, and 100 bilayers **(e)**.

**Figure 8:**
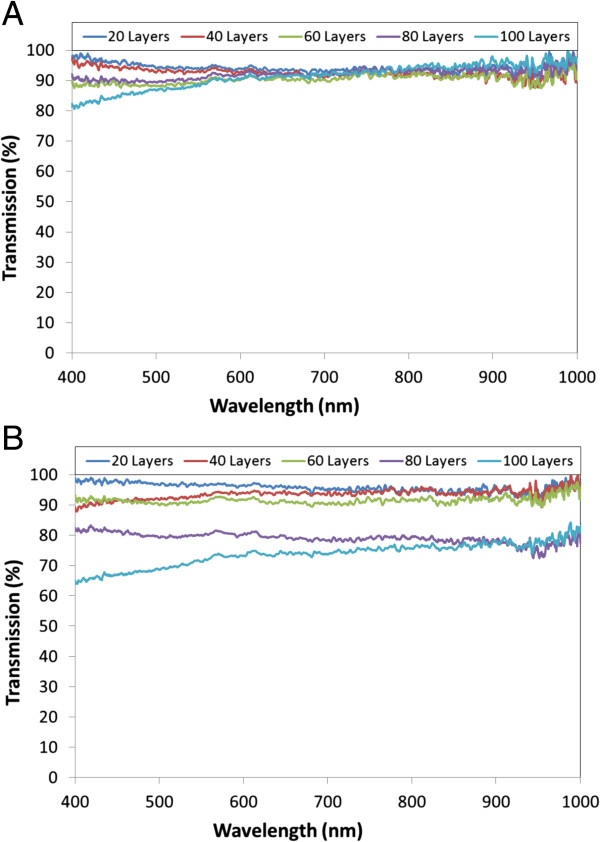
**Roughness RMS registered for the sprayed glass slides.** The left vertical axe is applied for the 10^-3^ M solutions and the right vertical axe for the 10^-4^ M ones.

**Figure 9:**
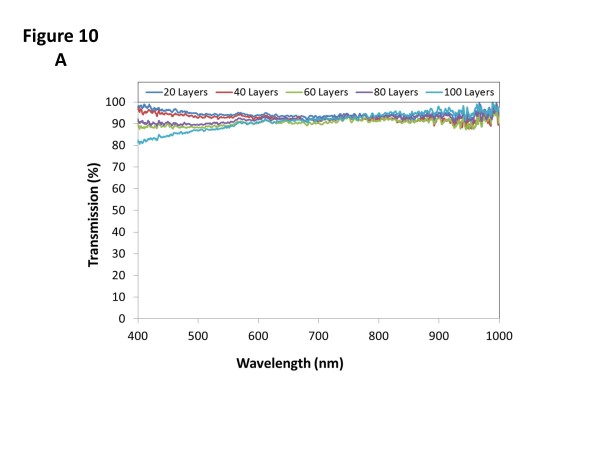
**Thickness recorded for the sprayed glass slides.** The left vertical axe is applied for the 10^-3^ M solutions and the right vertical axe for the 10^-4^ M ones.

Up to ten glass slides were coated by spray-assisted LbL to study the same parameters analyzed before for the LbL dip coating, five slides with 10^-4^ M solutions and the other ones with 10^-3^ M. The AFM images registered for the 10^-4^ M mixtures are shown in Figure 6. The films are also islandlike, showing an uniform pattern along the image in each case: the size of the island increases with the number of bilayers. Again, it denotes an increase of the roughness: actually, it goes from 4 nm RMS for 20 bilayers up to 50 nm RMS when 100 bilayers are deposited. In the case of the 10^-3^ M solutions, the AFM images can be observed in Figure 7: for 20 bilayers, the surface looks granular, but contrarily to the phenomena observed for the other coatings (films prepared with 10^-4^ M solutions), now as the number of sprayed bilayers increases, this appearance gets diffused. As a consequence, the roughness of the films prepared by spray-assisted LbL with the 10^-3^ M solutions decreases as the nanofilm grows, which is expected from LbL depositions [25], down to 1.23 nm RMS when 100 bilayers are deposited. The roughness obtained for both concentrations is displayed in Figure 8: the results from the nanoconstructions prepared with 10^-3^ M remark the decreasing roughness as the film increases, whereas the 10^-4^ M films show a monotonically increasing growth, confirming the surprising results reported by Decher et al. [23]. The thickness of the films are plotted in Figure 9: the values obtained with 10^-3^ M approximately double the ones registered with 10^-4^ M due to the lower concentration.

The contact angle measured for the 10^-4^ M prepared films falls to near 0 with 60 bilayers or more, highlighting the effect of the increasing roughness; on the contrary, for the films prepared with 10^-3^ M solutions, the contact angle remains above 30°, so they cannot be considered superhydrophilic.

**Figure 10:**
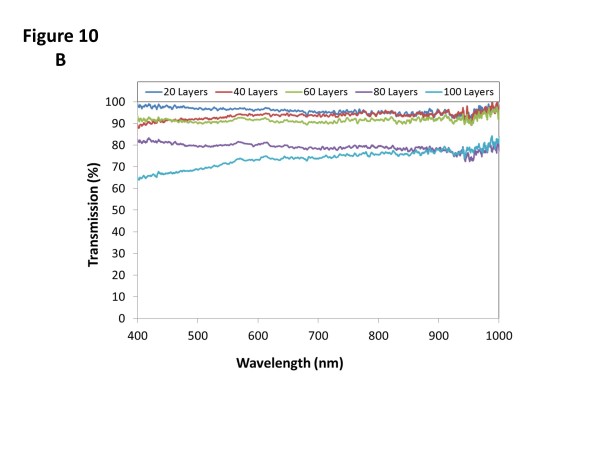
**Transmission spectra of films developed by spraying approach.** Transmission spectra measured for the films developed by spraying approach with the 10^-4^ M solutions **(a)** and the 10^-3^ M mixtures **(b)**.

The transmittance spectra registered for the different cases are plotted in Figure 10. For the first set of films (10^-4^ M), the optical transmittance is around 90%; only in the case of the thickest film that this value falls below 90% from 400 to 600 nm. The other set of films also shows a high-transmission spectra, above 90% with 60 bilayers or less and higher than 65% for the other two cases. The lower transmittance is a consequence of the higher thickness produced by the more concentrated solutions.

Results reported in this section are summarized in Table 2.

**Table 2:** Characterization of the films prepared using spraying approach

**Number of bilayers**	**Roughness**				**Thickness**				**Contact angle**			
	**10**^**-4**^ **M**		**10**^**-3**^ **M**		**10**^**-4**^ **M**		**10**^**-3**^ **M**		**10**^**-4**^ **M**		**10**^**-3**^ **M**	
	**μ**	**σ**	**μ**	**σ**	**μ**	**σ**	**μ**	**σ**	**μ**	**σ**	**μ**	**σ**
20	4.07	1.38	14.05	0.66	39.23	2.58	64.6	0.14	21.6	1.41	32.48	8.05
40	16.58	3.12	8.78	0.79	81.23	13.55	155	6.36	8.15	0.97	91	5.89
60	34.13	0.58	4.2	0.34	114.39	10.92	264.33	8.14	0	0	45.45	3.67
80	30	1.56	2.78	0.56	236.97	4.73	425.33	8.49	0	0	59.45	6.92
100	50.87	7.17	1.23	0.05	284.6	7.31	590.67	15.56	0	0	37.03	4.78

## Conclusions

In light of the results reported, both the polymeric concentrations and the deposition method (dipping or spraying) affect the growth of the nanofilms. The roughness obtained with the dipped slides is higher than the registered one with the sprayed substrates; on the other hand, the optical transmittance is lower as a consequence of the greater thickness obtained with the dipped slides. Moreover, in all cases but in the one with 10^-3^ M of sprayed solutions, the roughness is increased as the number of bilayers grows, which is an unexpected behavior in LbL films. It is also remarkable that the concentrations used here are lower than the ones typically studied in the literature, around 10^-2^ M [27]. The thickness and roughness observed using the dipping approach are higher than the ones registered with the sprayed slides: these differences have been observed in previous works [22]. The best results in terms of a superhydrophilic behavior are obtained with 10^-3^ M dipping solutions and with 10^-4^ M spraying mixtures. On the other hand, the high optical transmittance registered with the 10^-4^ M of sprayed solutions, even when 100 bilayers are deposited, points to its potential use in applications where superhydrophilic and transparent surface are required.

The use of inorganic short-chain polymers in LbL method shows that some assumed rules need to be redefined. In this work, it has been demonstrated that the roughness of nanofilms can increase as the growing process goes on, depending on the concentration of the polymers used and also on the way the slides are exposed to the solutions (dipped or sprayed). The highest roughness is obtained when the slides are dipped into the highest concentration solutions, which was supposed to produce the lowest roughness. The thickness of the resulting films falls in the nanometric range so they could be used in applications where surfaces have to be functionalized. Optical transmittance is above 90% for the films prepared with the 10^-4^ M of sprayed solutions, which highlights its potential used for preparing superhydrophilic transparent films. The use of PSP offers other important advantages: as it is an inorganic polymer, it can yield to surfaces whose degradation is lower than the ones prepared with organic polymers. Therefore, this work enforces to keep on studying the effect of this kind of polymers in LbL nanostructures.

## Abbreviations

AFM: Atomic force microscope; LbL: Layer-by-layer; PAH: Poly(allylamine hydrochloride); PEI: Poly(ethylenimine); PSP: Poly(sodium phosphate).

## Competing interests

The authors declare that they have no competing interests.

## Authors’ contributions

CE participated in the experimental work and carried out the AFM images. He also collaborated in the planning of the experiment; he prepared the drafting of the manuscript as well. DLT developed the films with the different number of bilayers and deposition approaches. He also contributed with the draft of the paper. MH participated in the experimental work registering AFM images and also contributed to the draft of the manuscript. IRM supervised the design of the study. FJA led the design of the study and helped to draft the manuscript. All authors read and approved the final manuscript.

## Supplementary Material

Additional file 1 **Contact angles recorded for each film.** Images of the contact angles. Four slides are available:1st slide, 10^-4^ M dipped films; 2nd slide, 10^-3^ M dipped films; 3rd slide, 10^-4^ M sprayed films; 4th slide, 10^-3^ M sprayed films. Click here for file
