# Supplementary material for: Comparative study of layer-by-layer deposition techniques for poly(sodium phosphate) and poly(allylamine hydrochloride)
Source: Nanoscale Res Lett. 2013 Dec 20;8(1):539. doi: 10.1186/1556-276X-8-539 (PMC3878125; doi:10.1186/1556-276X-8-539)
Supplement: Additional file 1 — Contact angles recorded for each film. Images of the contact angles. Four slides are available:1st slide, 10-4 M dipped films; 2nd slide, 10-3 M dipped films; 3rd slide, 10-4 M sprayed films; 4th slide, 10-3 M sprayed films. [file 1556-276X-8-539-S1.pptx]

## Slide 1
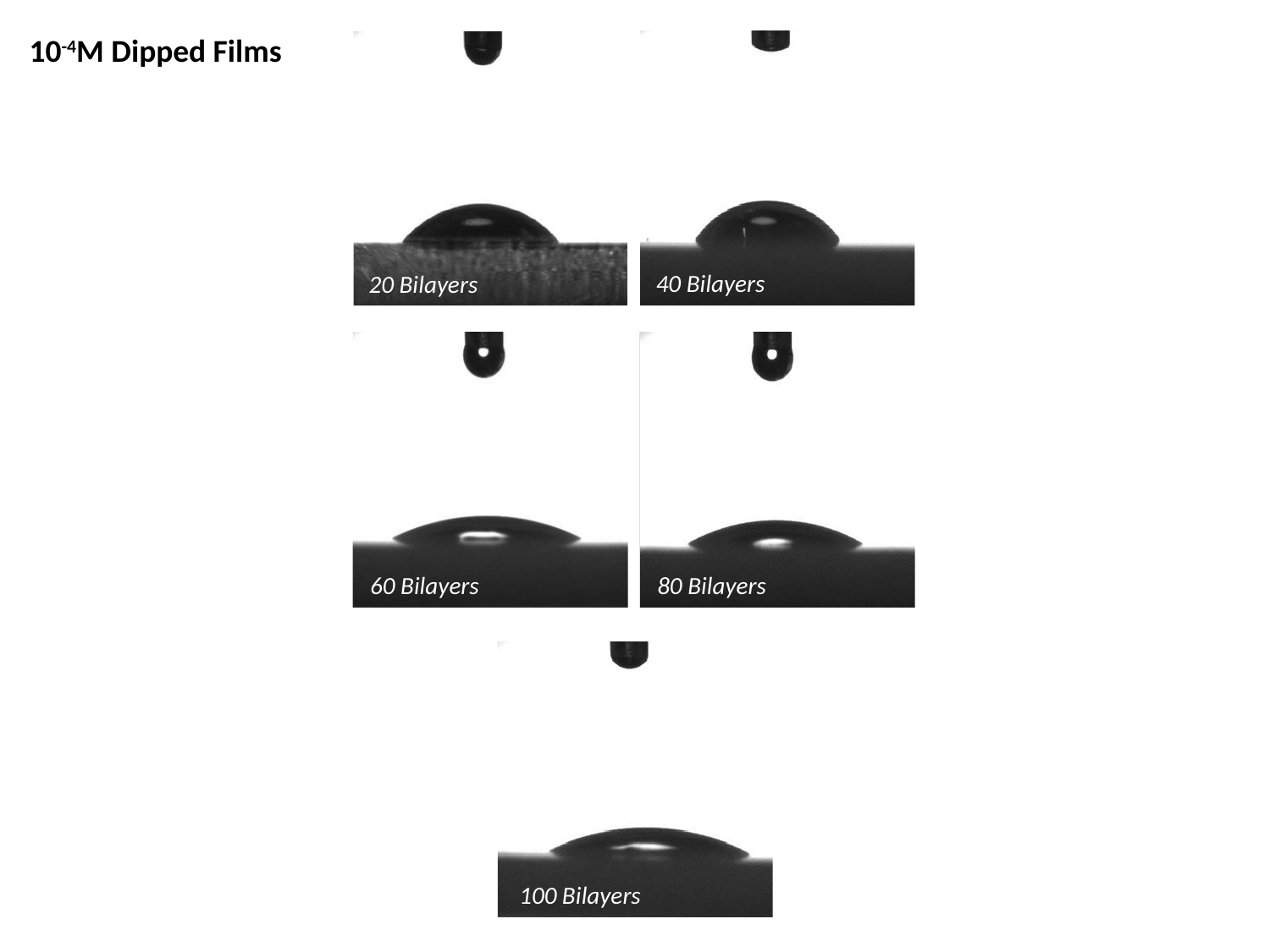

10-4M Dipped Films
40 Bilayers
20 Bilayers
80 Bilayers
60 Bilayers
100 Bilayers

## Slide 2
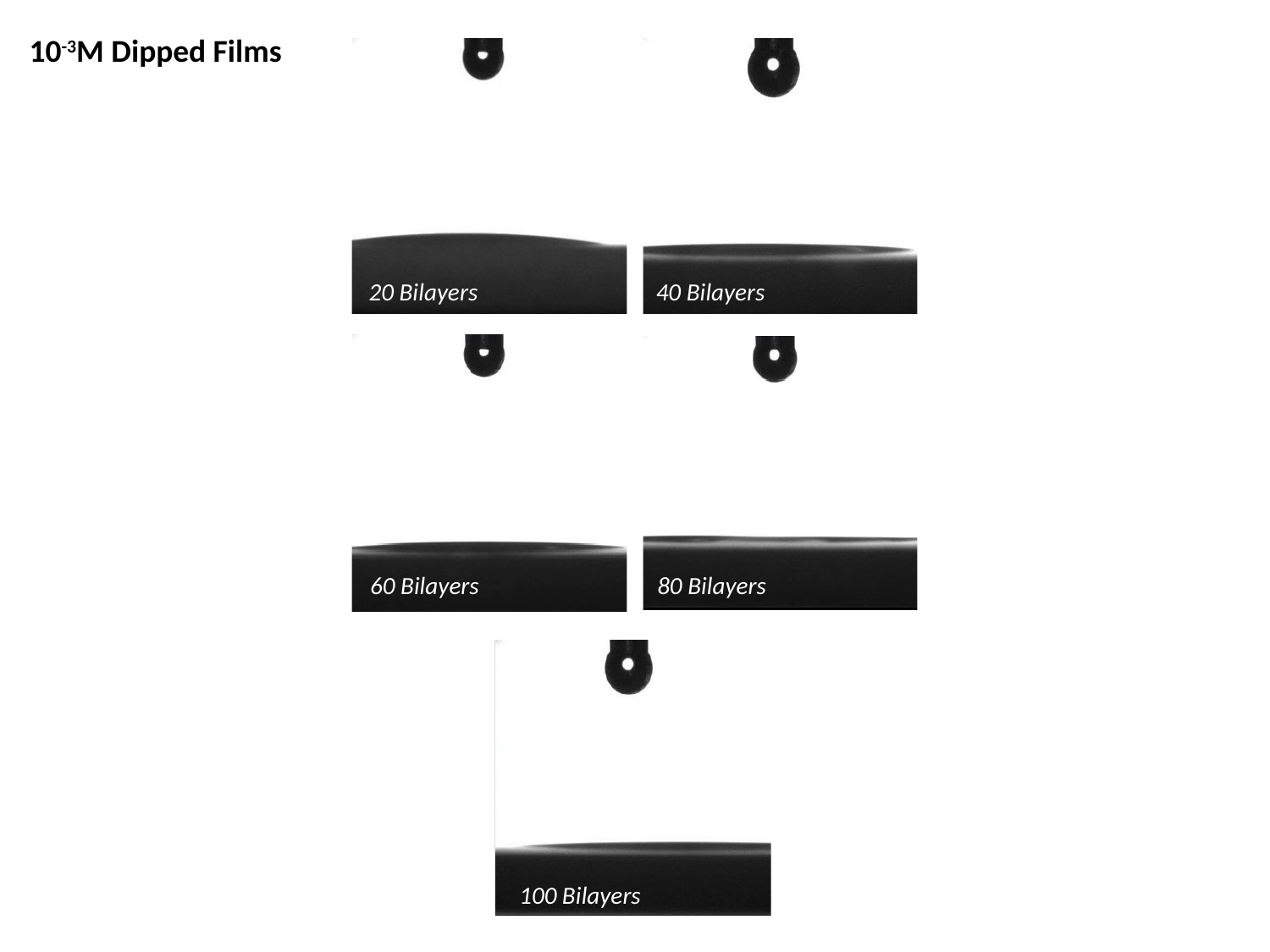

10-3M Dipped Films
40 Bilayers
20 Bilayers
80 Bilayers
60 Bilayers
100 Bilayers

## Slide 3
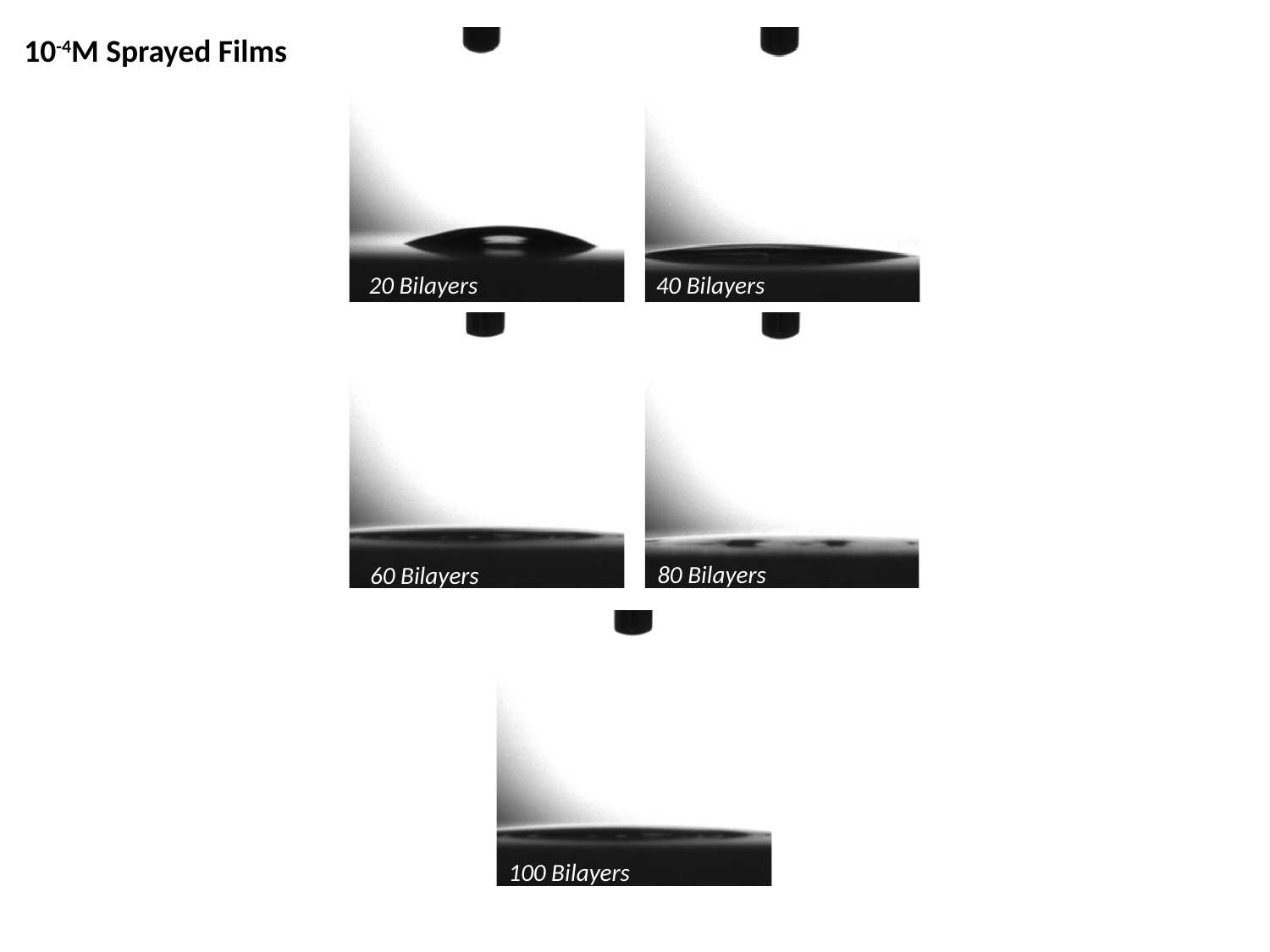

10-4M Sprayed Films
40 Bilayers
20 Bilayers
80 Bilayers
60 Bilayers
100 Bilayers

## Slide 4
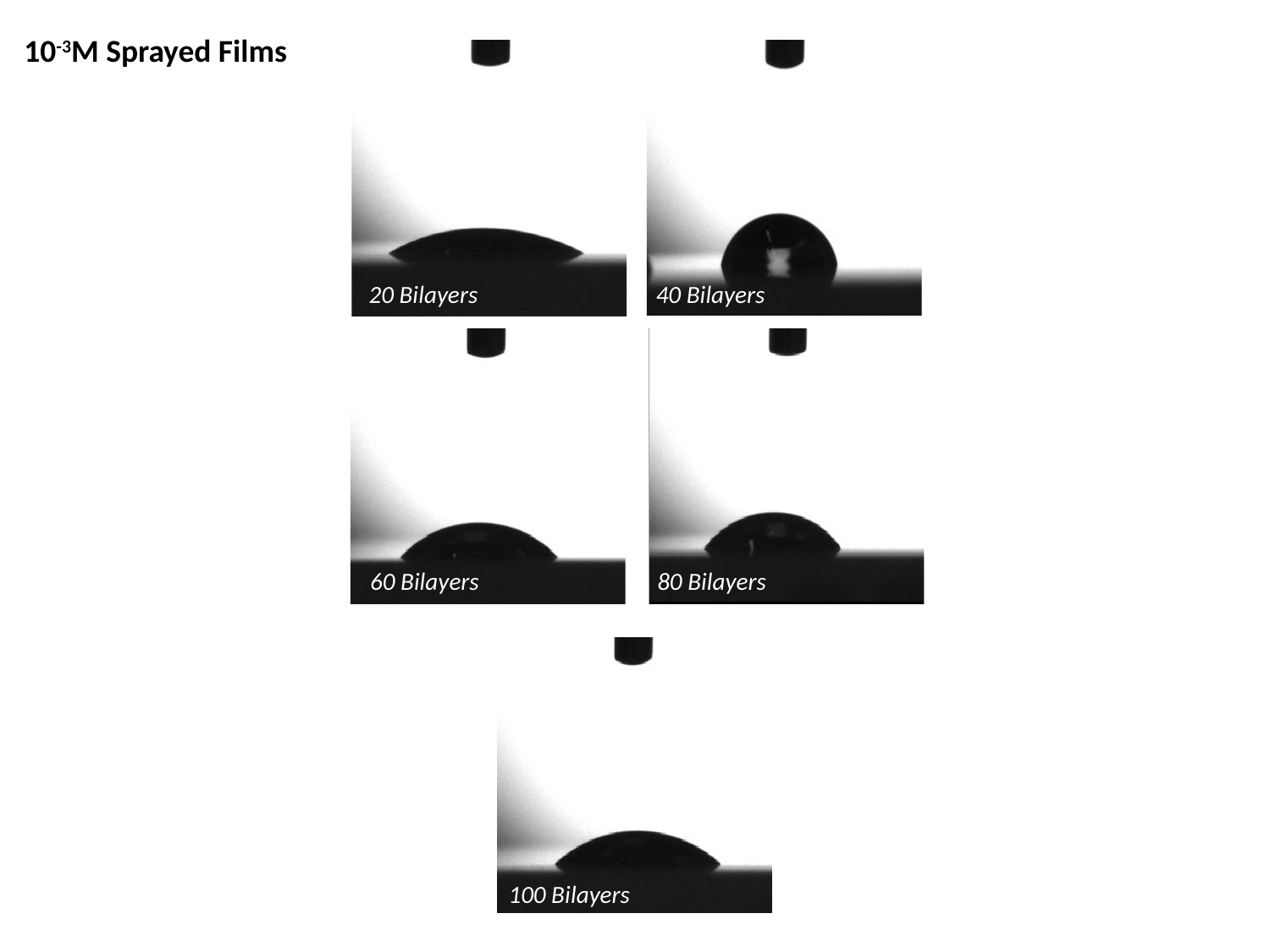

10-3M Sprayed Films
40 Bilayers
20 Bilayers
80 Bilayers
60 Bilayers
100 Bilayers
